# Autophagy Protects from Raddeanin A-Induced Apoptosis in SGC-7901 Human Gastric Cancer Cells

**DOI:** 10.1155/2016/9406758

**Published:** 2016-11-15

**Authors:** Yu-hao Teng, Jie-pin Li, Shen-lin Liu, Xi Zou, Liang-hua Fang, Jin-yong Zhou, Jian Wu, Song-yang Xi, Yan Chen, Ying-ying Zhang, Song Xu, Rui-ping Wang

**Affiliations:** ^1^Oncologic Department, Jiangsu Province Hospital of TCM, Nanjing, Jiangsu 210029, China; ^2^Nanjing University of Chinese Medicine, Nanjing, Jiangsu 210023, China

## Abstract

Raddeanin A (RA) is an extractive from* Anemone raddeana Regel*, a traditional Chinese medicine. The aim of this study is to assess the efficacy of RA against human gastric cancer (GC) cells (SGC-7901) and explore its mechanism. MTT assay showed that RA inhibition of proliferation of SGC-7901 cells increased in a dose-dependent manner. Flow cytometry analysis and Hoechst 33258 staining showed that RA induced apoptosis on SGC-7901 cells. Meanwhile, it induced autophagy. Western blotting analysis showed that the RA induces apoptosis and autophagy by activating p38 MAPK pathway and inhibiting mTOR pathway. Further studies showed that autophagy inhibition could protect from RA-induced apoptosis in SGC-7901 cells. In conclusion, RA can induce SGC-7901 cell apoptosis and autophagy by activating p38 MAPK pathway. And autophagy can protect SGC-7901 cells from apoptosis induced by RA.

## 1. Introduction

Gastric cancer (GC) is the third leading cause of cancer death worldwide. More than 50% of cases occur in Eastern Asia, while the highest estimated mortality rates are also in Eastern Asia [1]. Therefore, to improve the treatment level of GC in Eastern Asia is an urgent problem to be solved. However, GC is not sensitive to radiotherapy and chemotherapy. Traditional Chinese medicine (TCM) could be an important supplement. However, convincing evidence must be obtained and confirmed by high-quality trials in future studies [2].

Raddeanin A (RA) is an oleanane-type triterpenoid saponin extracted from* Anemone raddeana Regel*, a traditional Chinese medicine. Studies have shown that RA inhibits the growth of liver cancer and colorectal cancer [3, 4].

Autophagy, as another kind of programmed cell death (PCD), which is different from apoptosis, is a hot spot in cancer research in recent years. Autophagy represents an important cell-physiologic response that, like apoptosis, normally operates at low, basal levels in cells but can be strongly induced in certain states of cellular stress. Of note, recent research has revealed intersections between the regulatory circuits governing autophagy, apoptosis, and cellular homeostasis. But when and how autophagy causes the cancer cells' survival or death has always haunted us. In our preliminary studies, RA can induce stomach cancer cell apoptosis [5] and autophagy. As autophagy and apoptosis are two main ways of programmed cell death, we therefore assume that RA might induce both of them in stomach cancer. Furthermore, we want to know what role autophagy plays in the process of apoptosis induced by RA.

## 2. Materials and Methods

### 2.1. Drugs and Antibodies

RA was purchased from China National Institute for the Control of Pharmaceutical and Biological Products (purity > 99%). RA was dissolved in dimethyl sulfoxide (DMSO) as a stock solution (50 mM) stored at −20°C. 3-(4,5-Dimethylthiazol-2-yl)-2,5-diphenyltetrazolium bromide (MTT) and hydroxychloroquine sulfate (HCQ) were bought from Sigma Chemical Company (St. Louis, MO, USA). Annexin V conjugated to fluorescein-isothiocyanate (Annexin V-FITC) Apoptosis Detection kit was purchased from KeyGen Biotech. Co., Ltd. (Nanjing, China). Rapamycin (Rap) and rabbit anti-human monoclonal antibodies against BAX, Bcl-2, Bcl-XL, cleaved-PARP, RAS, p-p38, p-ERK, mTOR, p-mTOR, Beclin-1, ATG3, ATG5, ATG7, LC3b, and *β*-actin were purchased from Cell Signaling Technology (Beverly, MA, USA). Fluorescein-conjugated secondary antibodies were purchased from Odyssey (LI-COR, Belfast, ME, USA). All the other chemicals used in the experiment were of the highest purity grade available.

### 2.2. Cell Line and Culture

Human GC cell line SGC-7901 was offered by Type Culture Collection, Chinese Academy of Sciences (Shanghai, China), and cultured in RPMI-1640 medium (HyClone, Thermo Scientific, USA) containing 10% fetal bovine serum (FBS; HyClone, Thermo Scientific, USA) in a humidified incubator with 5% CO_2_ at 37°C.

### 2.3. MTT Assay

Cells in logarithmic growth phase were firstly seeded in 96-well culture plates at 6 × 10^3^ cells/well. Following treatment with drugs, MTT was added. Cells were then solubilized in DMSO. Absorbance was detected at 490 nm by using ELx800 microplate reader (BioTek, Winooski, VT). The same experiment was repeated 3 times, according to the following formula: inhibition rate = (1 − OD_experiment_/OD_control_) × 100%.

### 2.4. Cell Apoptosis Assay

The cells were seeded in 6-well plates and disposed with drugs for 12 h and then harvested by trypsinization, washed twice by cold phosphate buffer saline (PBS), and resuspended in 500 *μ*L binding buffer, to which 5 *μ*L Annexin V-FITC and 5 *μ*L Propidium Iodide (PI) were added. The cells were gently vortexed and incubated for 15 min at room temperature in the dark and then analyzed by flow cytometry (FCM). The same experiment was repeated 3 times.

### 2.5. Hoechst 33258 Staining

Cells were fixed with 4% paraformaldehyde for 30 min after being treated with drugs at room temperature and washed once with PBS. They were incubated in Hoechst 33258 (50 ng/mL) for 30 min and then washed with PBS. Apoptotic cells were identified by the condensation and fragmentation of their nuclei and photographed by a Zeiss Axioplan 2 fluorescence microscope (Jena, Germany). The same experiment was repeated 3 times.

### 2.6. Western Blotting Analysis

Cells were lysed in RIPA buffer in an ice bath for 20 min and centrifuged at 12,000 g for 20 min at 4°C. The supernatant was stored at −80°C until analyses. Protein concentration was measured by BCA method. An equal amount of proteins was loaded into 10% or 12% SDS-polyacrylamide gel for electrophoresis and transferred by electroblotting to polyvinylidene difluoride membrane (Millipore, Boston, MA, USA), which was blocked with 5% BSA, and then incubated with the indicated primary antibodies against BAX, Bcl-2, Bcl-XL, cleaved-PARP, RAS, p-p38, p-ERK, mTOR, p-mTOR, Beclin-1, ATG3, ATG5, ATG7, LC3b, and *β*-actin (at a dilution of 1 : 1,000) at 4°C overnight. After being washed by Tris Buffered Saline with Tween-20 (TBST) three times (5 min/time), secondary fluorescent antibody (1 : 5000 dilutions) was added to the membrane at room temperature for 1 h. After the membranes were washed for 5 min three times with TBST, the signals intensity of the membranes was detected by Odyssey (LI-COR, Belfast, ME, USA); *β*-actin was used as the loading control. The same experiment was repeated 3 times.

### 2.7. Electron Microscope Analysis

For observation under transmission electron microscope (TEM), cells washed with PBS were pelleted and the cell pellet was fixed in 2.5% glutaric dialdehyde and 1% osmic acid for 2 h. After the wash, the pellet was dehydrated and embedded in epoxy resin. The ultrathin sections (45 nm) were stained with lead acetate, and the stained sections were observed under JEOL-1010 electron microscope [6].

### 2.8. Statistical Analysis

All data were presented as means ± standard deviation (SD). Statistical analysis was performed by SPSS 17.0 software with one-way ANOVA followed by Dunnett's test to compare the treatment groups and the control group.

## 3. Results

### 3.1. RA Induces Apoptosis

**Figure 1:**
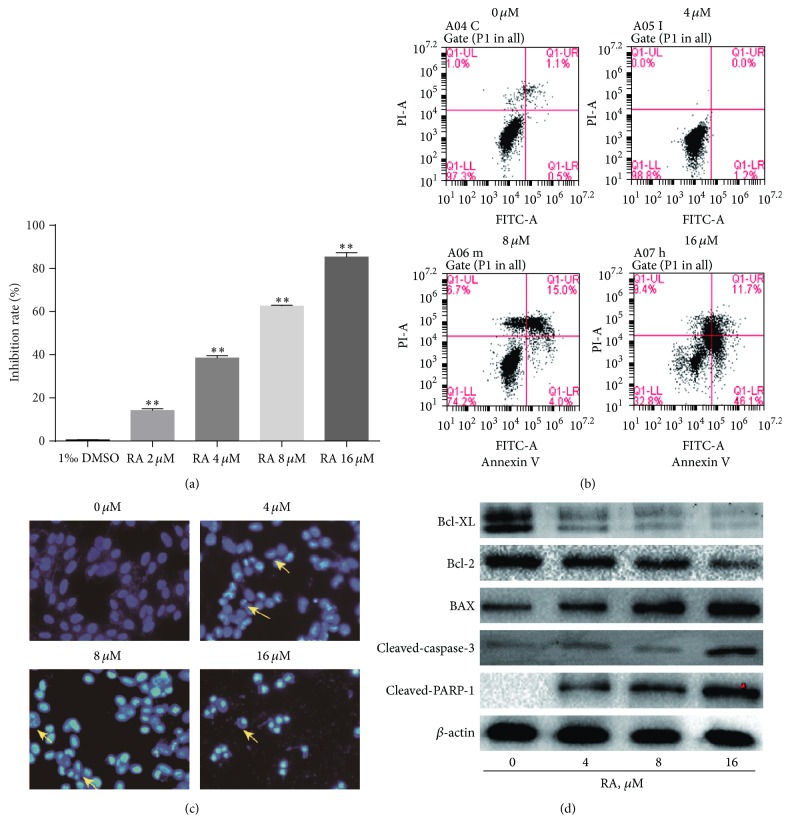
(a) Effects of RA on cell inhibition. Cells were treated with different concentrations of RA for 12 h. The bars indicate standard errors. The asterisk indicates a significant increase in the inhibition rate between groups treated with RA or not. Data are expressed as mean ± SD of three experiments (^*∗∗*^ *P* < 0.05). (b) Effects of RA on cell apoptosis. SGC-7901 cells were treated with different concentrations of RA for 12 h and harvested by trypsinization and centrifugation and then analyzed by FCM after staining with Annexin V-FITC and PI. Results shown are of an experiment representative of apoptosis. Q1-UL showed that cells were undergoing necrosis, and Q1-UR showed that cells were at the end stage of apoptosis. Q1-LL showed that cells were viable or there was no measurable apoptosis. Q1-LR showed that cells were undergoing apoptosis. (c) The morphologic change was assessed by fluorescence microscopy. Cells were fixed with 4% paraformaldehyde for 30 min after being treated with different concentrations of RA for 12 h at room temperature and washed once with PBS. Hoechst 33258 (50 ng/mL) was incubated for 30 min and then washed with PBS. Apoptotic cells were identified by the condensation and fragmentation of their nuclei and photographed by a Zeiss Axioplan 2 fluorescence microscope (400x). (d) The levels of apoptosis related proteins were tested by western blotting analysis. After cells were treated with 0, 4, 8, and 16 *μ*M of RA for 12 h, the protein levels of Bcl-XL, Bcl-2, BAX, cleaved-caspase-3, and cleaved-PARP were determined by western blotting.

It is well known that resisting cell death is one of the characteristics of cancer cells. MTT assay was used to investigate the growth inhibitory effect of RA on SGC-7901 cells. After the cells were treated with 0, 2, 4, 8, and 16 *μ*M of RA for 12 h, the effect of RA on inhibition rate of the cells was observed via the MTT assay. As the result seen from Figure 1(a), RA can efficiently inhibit proliferation of SGC-7901 cells. When the concentration of RA reached 16 *μ*M, the inhibition rates rose to 85.31%  ±  1.09%, respectively. These results are similar to our previous research results [5]. Based on this result, concentrations of 4, 8, and 16 *μ*M were set for subsequent studies.

Evading apoptosis is the primary means of cancer cells to escape death. Therefore, induction of apoptosis is an important way to kill cancer cells. In this study, we further tested whether RA induced SGC-7901 cells apoptosis. We evaluated it by using FCM and observed the nuclear morphological changes of cells by using Hoechst 33258 staining.

After SGC-7901 cells were treated with 0, 4, 8, and 16 *μ*M of RA for 12 h, both early and late apoptotic rates of the cells were increased in a dose-dependent manner (Figure 1(b)). When the concentration of RA reached 16 *μ*M, the early and late apoptotic rates rose to 46.1% and 11.7%, respectively. The morphologic change was assessed by fluorescence microscopy after being stained with Hoechst 33258. For the treated cells, we observed small, fragmented, and condensed nuclei with typical apoptotic morphology in contrast with normal symmetrical, blue nuclei (Figure 1(c)).

After cells were treated with 0, 4, 8, and 16 *μ*M of RA for 12 h, we found that the levels of cleaved-PARP increased in a dose-dependent manner. This confirmed that RA induced apoptosis at a molecular level. Furthermore, we found that the levels of BAX and cleaved-caspase-3 increased, while those of Bcl-2 and Bcl-XL decreased in a dose-dependent manner (Figure 1(c)).

### 3.2. RA Induces Autophagy

As an important way of type II programmed cell death (PCD), autophagy has been paid more and more attention. Here, we tested whether RA could induce autophagy in SGC-7901 cells.

**Figure 2:**
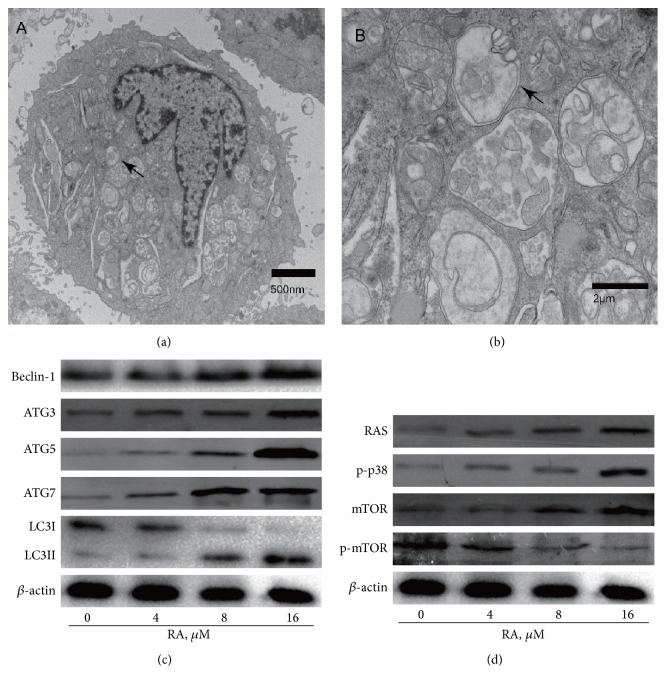
(a) After being treated with 8 *μ*M of RA for 12 h, cells were harvested and observed by TEM. TEM of autophagosome in SGC-7901 cells treated with RA (10,000x). (b) The enlarged high resolution images of the square areas were shown (50,000x). The arrow points to a typical autophagosome with a bilayer membrane structure. (c) Western blotting analysis showed the levels of autophagy related proteins. After cells were treated with 0, 4, 8, and 16 *μ*M of RA for 12 h, the protein levels of LC3, Beclin-1, ATG3, ATG5, and ATG7 were determined by western blotting. (d) Western blotting analysis showed the levels of MAPK pathway and mTOR pathway. After cells were treated with 0, 4, 8, and 16 *μ*M of RA for 12 h, the protein levels of p-p38, p-ERK, mTOR, and p-mTOR were determined by western blotting.

After being treated with 8 *μ*M of RA for 12 h, cells were harvested and observed by TEM. And we found a typical autophagosome with a bilayer membrane structure (Figure 2(b)). In order to further confirm it, autophagy associated proteins were detected by western blotting analysis. The conversion of LC3I to LC3II reflects the occurrence of autophagy. After cells were treated with 0, 4, 8, and 16 *μ*M of RA for 12 h, we found that the levels of LC3II increased, while those of LC3I decreased in a dose-dependent manner (Figure 2(c)). Meanwhile, the levels of Beclin-1, ATG3, ATG5, and ATG7 increased in a dose-dependent manner (Figure 2(c)).

### 3.3. RA Induces Apoptosis and Autophagy by Activating p38 MAPK Pathway

p38 MAPK pathway has been proved to be associated with induction of apoptosis [7, 8] and autophagy [9]. We therefore speculated that RA might induce apoptosis and autophagy in SGC-7901 cells by suppressing the p38 MAPK signaling pathway. To test this hypothesis, cells were treated with 0, 4, 8, and 16 *μ*M of RA for 12 h, and it was found that the levels of p-p38 increased in a dose-dependent manner (Figure 2(c)). Another protein ERK in the MAPK pathway is also activated (Figure 2(c)).

Two relatively well-established pathways that regulate autophagy contain the mTOR and IP3 cascades. Inhibition of mTOR activity or a decrease of IP3 level can induce autophagy, respectively [10]. In this study, we found that the mTOR pathway was suppressed by RA (Figure 2(c)).

### 3.4. Inhibiting Autophagy Promotes RA-Induced Apoptosis

**Figure 3:**
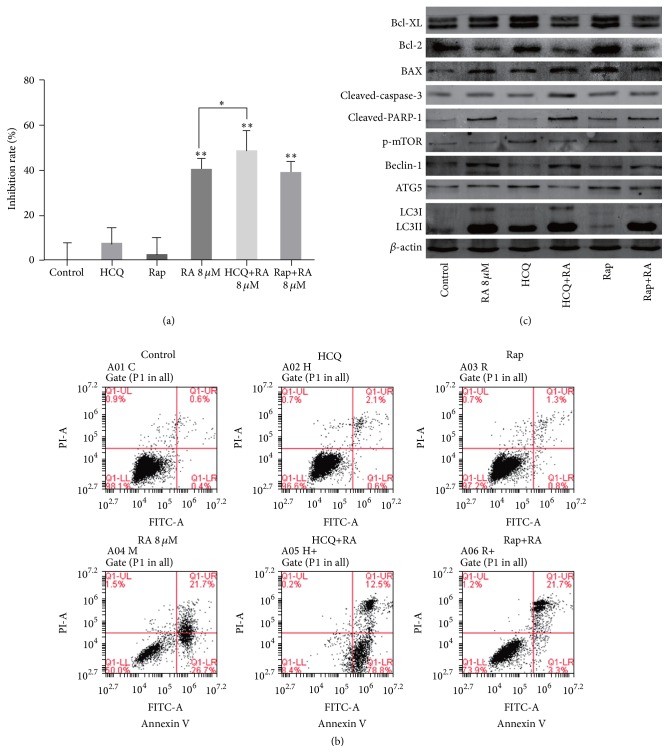
(a) Cell inhibition rates were measured by MTT assay, after inhibiting or inducing autophagy. HCQ (1 *μ*M) was added as autophagy inhibitor, while Rap (100 nM) was used as autophagy inducer. The bars indicate standard errors. The asterisk indicates a significant increase in the inhibition rate between groups. Data are expressed as mean ± SD of three experiments (^*∗*^ *P* < 0.05, ^*∗∗*^ *P* < 0.01). (b) Cell apoptosis rates were measured by FCM, after inhibiting or inducing autophagy. Results shown are of an experiment representative of apoptosis. Q1-UL showed that cells were undergoing necrosis, and Q1-UR showed that cells were at the end stage of apoptosis. Q1-LL showed that cells were viable or there was no measurable apoptosis. Q1-LR showed that cells were undergoing apoptosis. (c) Western blotting analysis showed the levels of apoptosis and autophagy related proteins, after inhibiting or inducing autophagy. The protein levels of Bcl-XL, Bcl-2, BAX, cleaved-caspase-3, cleaved-PARP, Beclin-1, ATG5, LC3, and p-mTOR were determined by western blotting.

As an important way of type II PCD, autophagy has also been reported as a survival mechanism in many tumor cells, allowing them to escape from apoptotic death in response to metabolic crisis. We wanted to know what role autophagy plays in the process of RA antitumor. HCQ was reported as an autophagy inhibitor, while Rap was reported as an autophagy inducer. Based on that, we first examined the rate of cell proliferation, while inhibiting or inducting autophagy. HCQ (1 *μ*M) was added as autophagy inhibitor, while Rap (100 nM) was used as autophagy inducer. We found that inhibition of autophagy can enhance RA inhibition of cell proliferation (*P* < 0.05), while induction of autophagy can slightly reduce (*P* > 0.05) (Figure 3(a)). Furthermore, Annexin V/PI double staining prompt apoptosis rate showed similar changes (Figure 3(b)). Therefore, we hypothesized that autophagy can protect cells from apoptosis in this case.

To further confirm this inference, we examined the related protein (Figure 3(c)). And we found that using Rap alone can inhibit p-mTOR, thereby promoting autophagy and increasing the expression of autophagy related protein. However, if HCQ is used alone, the expression of these proteins will not change significantly. Increased expression of LC3 is due to the inhibition of HCQ on autophagic lysosomal degradation. Compared with the RA group, RA combined with HCQ can weaken the upregulation expression on the autophagy related proteins and further increase the apoptosis related protein expression. Meanwhile, RA combined with Rap, these changes turn to be in the opposite (Figure 3(c)). These results suggested that HCQ can inhibit autophagy, while Rap can induce autophagy in SGC-7901 cells. Furthermore, HCQ can induce apoptosis of SGC-7901 cells by inhibiting autophagy, which is the opposite of Rap.

## 4. Discussion

In recent years, with the progress of treatment, the survival rate of GC patients is increasing. However, many patients still relapse after surgery, radiotherapy, and chemotherapy. Thus, preventing recurrence and metastasis of GC has become one of the focuses of the present studies. Resisting cell death and self-sufficiency in growth signals are intrinsic characteristics of all kinds of carcinoma [11], including GC. Based on these, moderately differentiated GC cell line (SGC-7901) was selected for the present research.

RA has been used to inhibit growth and induce apoptosis of cancer cells as natural bioactive substance in several studies. Its potential in developing effective anticancer and chemopreventive approaches has been focused on in previous studies [3–5]. However, the research on the mechanism of RA against GC is still rare. Thus, we explored the effect of RA on apoptosis and autophagy of GC cells and the related molecular mechanism. We also explored the role of autophagy in RA-induced apoptosis.

Resisting apoptosis is the principal mechanism of tumor cell to resisting death and also is an important cutting-in point for the development of anticancer drugs. In the present study, we applied Annexin V-FITC/PI double staining FCM (Figure 1(b)) and Hoechst staining (Figure 1(c)) to investigate effects of RA on the apoptosis of human GC cells. The results indicated that RA induced apoptosis on both early and late stages.

Apoptosis can be triggered by two signaling pathways, the extrinsic pathway (death receptor pathway) and the intrinsic pathway (the mitochondrial pathway) [12]. Poly(ADP-ribose) polymerase (PARP) is a key signaling nuclear protein involved in apoptosis. Activated caspase protein cuts it into cleavage of poly(ADP-ribose) polymerase (c-PARP) which is a symbol progress in apoptosis [13, 14]. To further explore the molecular mechanism, western blotting analysis was used (Figure 1(d)). Then, we found that the level of PARP protein decreased in a dose-dependent manner, while the c-PARP increased. Bcl-2 family played a significant role in apoptosis [15]. Particularly, the stoichiometries of BAX (proapoptotic gene) and Bcl-2 (antiapoptotic gene) are influential factors for the downstream activation of caspase protein [16, 17]. Therefore, we continued to detect the expression of Bcl-2 family. We found that the ratio of BAX/Bcl-2 rose with dose increasing. These results suggest that RA can regulate the ratio of BAX/Bcl-2 to induce apoptosis in human GC cells.

Autophagy represents an important cell-physiologic response that, like apoptosis, normally operates at low, basal levels in cells but can be strongly induced in certain states of cellular stress, such as nutrient deficiency, lack of growth factors, oxidative stress, hypoxia, radiation, and chemotherapy. However, excessive autophagy can also lead to a non-caspase-dependent cell death [18, 19].

In the present study, we used TEM to observe effects of RA on the autophagy of human GC cells. We fortunately observed autophagosome inside the cell treated with RA (Figures 2(a) and 2(b)). And we found that the levels of autophagy related proteins increased in a dose-dependent manner (Figure 2(c)). These results show that RA can increase the expression of autophagy related proteins to promote autophagy in human GC cells.

Recent research has revealed intersections between the regulatory circuits governing autophagy and apoptosis. However, the relationship between autophagy and apoptosis confused us a lot. Apoptosis and autophagy are not independent processes, given that a complex cross talk between them has been depicted, leading to the notion that they can be triggered by common upstream signals and share molecular switches [20]. Beclin-1 and Bcl-2 family members represent the best example: Bcl-2 and Bcl-XL inhibit Beclin-1 by binding to it through the Beclin-1 BH3 domain. JNK-1 can promote the dissociation of Bcl-2/Beclin-1 complex. Subsequently, Bcl-2 exerts its antiapoptotic effect, and Beclin-1 plays its role in promoting autophagy. In most reports, autophagy can protect cancer cells from apoptosis, by providing nutrition, inducing resistance [21, 22], and so forth.

In the present study, we applied MTT assay (Figure 3(a)) and FCM assay (Figure 3(b)) to detect the effects of RA on SGC-7901 cells, after inhibition or induction of autophagy. Western blotting analysis subjected that HCQ can induce apoptosis of SGC-7901 cells by inhibiting autophagy, which is the opposite of Rap (Figure 3(c)). These results indicated that autophagy can protect SGC-7901 cells from RA-induced apoptosis.

Self-sufficiency in growth signals is another character of tumor cells. As one of the most important intracellular pathways, MAPK pathway is frequently activated in a number of cancers and is responsible for the resistance of cell growth, autophagy [9], apoptosis [8, 23], metabolism, and invasion [24]. In the present research, it is discovered that RA could effectively activate p38 MAPK pathway (Figure 2(d)).

In conclusion, RA can induce SGC-7901 cell apoptosis and autophagy by activating p38 MAPK pathway. And autophagy can protect SGC-7901 cells from apoptosis induced by RA. These findings provide an experimental basis for RA as a drug for GC and with the autophagy inhibitor combined can improve its efficacy, but more basic research is needed to verify the antitumor activity of RA.
